# Reply to Doboszewska et al.: The many faces of G-protein-coupled receptor 39

**DOI:** 10.1073/pnas.2308643120

**Published:** 2023-07-03

**Authors:** Jie-Mei Wang

**Affiliations:** ^a^Department of Pharmaceutical Sciences, Eugene Applebaum College of Pharmacy and Health Sciences, Wayne State University, Detroit, MI 48201; ^b^Centers for Molecular Medicine and Genetics, Wayne State University, Detroit, MI 48201; ^c^Karmanos Cancer Institute, Detroit, MI 48201

G-protein-coupled protein receptors (GPCRs, or GPRs) take up a large portion of the drug target market, but many GPRs remain “orphans” due to their unclear endogenous ligands or intracellular signals. GPR39, since it was first identified as a member of the ghrelin receptor family, is considered an orphan receptor (1), challenging its routes to clinical translation.

Our recent report suggests a mechanism for GPR39 to modulate angiogenesis through its constitutive and ligand-dependent activities in endothelial cells (ECs) because genetic GPR39 inhibition leads to augmented angiogenesis, while GPR39 high function, either by overexpression (without binding to ligand) or by agonist stimulation, suppresses the angiogenesis (2). Although we showed that the sonic hedgehog pathway is part of GPR39 intracellular signaling, the endogenous ligands of GPR39 are still unclear.

To date, only a limited number of endogenous ligands have been suggested for GPR39, such as zinc and eicosanoids, including 14,15-epoxyeicosatrienoic acid (14,15-EET) and 15-hydroxyeicosatetraenoic acid (15-HETE). However, significant discrepancies exist in potency at their physiologically relevant concentrations, partial agonisms/antagonisms, and tissue selectivity. Zinc is considered an activator and allosteric modulator for GPR39 in various tissues (3, 4). Recently, Doboszewskaa et al. proposed a working model for GPR39 as a receptor for 14,15-EET and 15-HETE in angiogenesis (5), based on recent reports showing that 15-HETE and 14,15-EET competitively act through GPR39 in microvascular smooth muscle cells (SMCs) (6) and that 15(S)-HETE promotes hypoxia-induced Rac1 activation in ECs (7). It, therefore, opens the discussion for eicosanoids as GPR39’s natural ligands. These findings will, in no doubt, warrant future investigations into the functions of these eicosanoids in regulating GPR39 signaling in ECs as outlined in the working model. One precaution is that the culture system with a relatively high concentration of these eicosanoids (at µM levels) and short exposure time (minutes) might need to compare with that at pathophysiologically relevant concentrations (at nM levels) and longer-term exposure (days). An existing example is that high glucose exposure requires days or weeks to recapture the EC dysfunctions seen in diabetic patients (8), while hours of high glucose increase EC angiogenic activity (9). In addition, whether increased EC angiogenesis reflects improved vascular health or is part of the maladaptive vascular processes needs careful interpretation. The latter might mediate the response to vascular stress, thereby promoting inflammation and oxidative stress in the vessel wall in the long term (10).

Adding to another layer of complexity is the divergent roles of GPR39 in various tissues and cell types. This is particularly true in metabolic and inflammatory disorders in which GPR39 manipulations have shown contradicting results in energy metabolism and vascular health (11). Recent technical advances using proximity-based labeling coupled with mass spectrometry might be a valuable tool to decipher GPR39’s spatial coexistence in subcellular compartments, upstream activators/inhibitors, and downstream targets. It will also help to quest endogenously ligands, especially if an autocrine mechanism for that ligand is suspected. Together with screening GPR39 ligands, these research endeavors will allow unique hypotheses and regimens to be tested pharmacologically.
